# Development of an Off‐Grid Solar‐Powered Autonomous Chemical Mini‐Plant for Producing Fine Chemicals

**DOI:** 10.1002/cssc.202102011

**Published:** 2021-11-08

**Authors:** Tom M. Masson, Stefan D. A. Zondag, Koen P. L. Kuijpers, Dario Cambié, Michael G. Debije, Timothy Noël

**Affiliations:** ^1^ Flow Chemistry Group van't Hoff Institute for Molecular Sciences (HIMS) Universiteit van Amsterdam (UvA) Science Park 904 1098 XH Amsterdam The Netherlands; ^2^ Department of Chemical Engineering and Chemistry Sustainable Process Engineering, Micro Flow Chemistry & Synthetic Methodology Eindhoven University of Technology Het Kranenveld, Bldg 14 – Helix 5600 MB Eindhoven The Netherlands; ^3^ Current address: Technology & Engineering Janssen R&D Turnhoutseweg 30 2340 Beerse Belgium; ^4^ Current address: Department of Biomolecular Systems Max Planck Institute of Colloids and Interfaces Am Mühlenberg 1 14476 Potsdam Germany; ^5^ Department of Chemical Engineering and Chemistry Stimuli-responsive Functional Materials & Devices Eindhoven University of Technology Groene Loper 3, Bldg 14 – Helix 5600 MB Eindhoven The Netherlands

**Keywords:** energy conversion, photocatalysis, photochemistry, solar energy, solar mini-plant

## Abstract

Photochemistry using inexhaustible solar energy is an eco‐friendly way to produce fine chemicals outside the typical laboratory or chemical plant environment. However, variations in solar irradiation conditions and the need for an external energy source to power electronic components limits the accessibility of this approach. In this work, a chemical solar‐driven “mini‐plant” centred around a scaled‐up luminescent solar concentrator photomicroreactor (LSC‐PM) was built. To account for the variations in solar irradiance at ground level and passing clouds, a responsive control system was designed that rapidly adapts the flow rate of the reagents to the light received by the reaction channels. Supplying the plant with solar panels, integrated into the module by placing it behind the LSC to utilize the transmitted fraction of the solar irradiation, allowed this setup to be self‐sufficient and fully operational off‐grid. Such a system can shine in isolated environments and in a distributed manufacturing world, allowing to decentralize the production of fine chemicals.

## Graphical abstract

**Going off‐grid**: An energy neutral scaled‐up luminescent solar concentrator photo‐microreactor (LSC‐PM) is used to perform solar photochemistry as an off‐grid chemical production platform. A control system ensures autonomous production of fine chemicals under fluctuating solar light irradiation and simulations provide insight for the implementation of the system around the world.

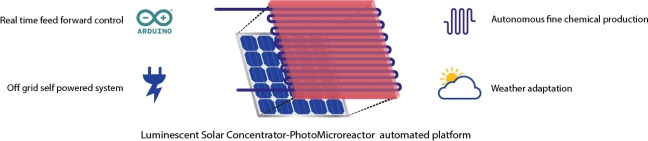

## Introduction

From Christopher Columbus to Neil Armstrong, humankind has always been drawn toward the exploration of new territories. Today, our robots are reaching the border of Mars and will keep on extending further.^[1]^ In those isolated and hostile lands, it becomes fundamental to have self‐sustaining systems to provide energy, food and medicine. These systems must cope with environmental fluctuations and be energetically independent. As chemists and chemical engineers, we aim at contributing to those explorations by creating synthesis systems that are fully independent and that can harvest energy directly from the environment.[^2^, ^3^]

The sun is a source of energy accessible to the entire planet, and serves as an ideal solution to power chemistry at isolated locations.[^4^, ^5^] In this regard, the recent improvements in the field of photoredox processes have greatly expanded the photochemical toolbox allowing harvesting of visible light wavelengths and enabling complex and previously elusive chemical bond transformations.[^6^, ^7^, ^8^] These tools can be progressively combined with solar chemistry, but fluctuating solar conditions often limit the attractiveness and reliable applicability of this field. To cope with these fluctuations and to ensure reliable production processes, direct control over the reaction and process parameters must be maintained.

Micro/milli‐flow technology presents itself as the better option to harvest solar light owing to the large exchange surface area and the possibilities of scaling.[^8^, ^9^, ^10^, ^11^, ^12^] As a recent invention from our laboratory, the combination of flow chemistry with inexpensive luminescent solar concentrators[^13^, ^14^, ^15^, ^16^] (LSCs) allows for enhanced, solar‐driven photochemical reactions.[^17^, ^18^] Herein, sunlight energy is collected, converted, concentrated and directed towards the reaction channels, maximizing the number of photons reaching the reaction mixture. However, we realized that a significant fraction of the solar spectrum remained unused (i. e., >600 nm).^[18]^ We surmised that these photons (up to 1100 nm)^[19]^ otherwise escaping the reactor can be collected with photovoltaic cells (PV) to produce electricity.^[20]^ This observation inspired us to make the first steps towards an off‐grid solar‐driven mini‐plant by integrating an LSC‐PM and a solar panel for energy production. The solar energy that is not used for chemical production can then be productively utilized to drive pumps, mass flow controllers, sensors and a regulation system that maintains constant chemical conversion during fluctuating irradiation.^[21]^ Consequently, this mini‐plant provides a steady flow of desired chemicals in a self‐sufficient manner without external energy supply, save for the sun. Through modelling and simulations, the optimal orientation of the reactor could be determined depending on the global location. With this data and the capability of the LSC‐PM to utilize both direct and diffuse light, solar tracking becomes unnecessary. Notably, this easy‐to‐build setup can be readily adapted to perform a large variety of photochemical transformations under controlled conditions.^[17]^

## Results and Discussion

### Design of the solar‐driven mini‐plant

The reactor is based on a 15 mL LSC‐PM (470×470×8 mm^3^) module. This reactor is doped with the commercial fluorescent dye Lumogen F Red 305 (BASF, ‘LR305’),^[22]^ which combines high fluorescent yields^[23]^ and excellent photostability^[24]^ to bring the luminescent properties to the polymer. The assembly of the panel is described in the Supporting Information, and adapted from a previously reported method by our group.^[17]^ The concentrated sunlight is guided towards the reaction channels and used to perform the photochemical transformations. The reactor is fed by an HPLC pump (Knauer Azura P4.1S) that introduces the reaction mixture inside the reactor channels. The incoming liquid flow is merged with an oxygen flow controlled by a mass flow controller (Bronkhorst, O_2_, max. 25 mL min^−1^, ‘MFC’).

To cope with fluctuating irradiation conditions, which are inevitable when using the sun as light source (e. g., passing clouds, day/ night cycles), a control system including a light sensor is attached to the edge of the LSC‐PM.^[17]^ The edge emission (EE) from the LSC lightguide is monitored in real‐time, and the data is used to adjust the pump‐driven flow rates of both oxygen and reagents to match the current incident light intensity following an experimentally established conversion correlation, maintaining constant production quality.

**Figure 1:**
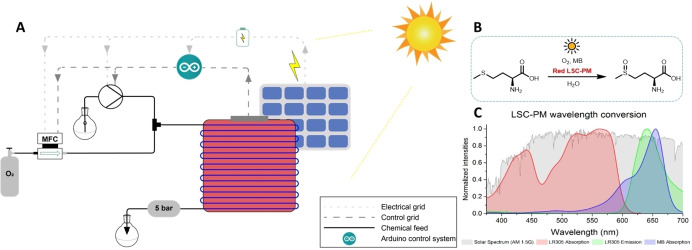
Working principle of the system. (A) Scheme of the solar mini‐plant showing the main components: the LSC‐PM, the external solar panel, the reactant feeds, MFC and the flow control system. (B) Oxidation of l‐methionine to l‐methionine sulfoxide using Methylene Blue (MB) as photocatalyst: 0.1 m l‐methionine, 1 mol% MB in H_2_O. (C) Wavelength conversion scheme LSC‐PM and Methylene Blue. The absorption (red area) and emission (green area) of the LR305 dye compared to the absorption spectrum of the MB photocatalyst (blue area). Superimposed on the spectra is the AM 1.5 solar spectrum. Reprinted with permission of Ref. [18]. Copyright 2016 John Wiley and Sons.

The electrical equipment of the mini‐plant is powered by solar panels charging a battery, where the battery acts as a power buffer when the solar irradiation fluctuates. The battery can deliver 5 V DC to supply the control system and 230 V AC to supply the MFC and the pump (see Figure 1A).

### Model reaction

The oxidation of l‐methionine to the corresponding sulfoxide (Figure 1B), previously already used for LSC‐PM applications,^[17]^ was chosen as a benchmark reaction, since having water as a solvent is easily scalable and safe to operate outdoors. Interestingly, methionine sulfoxide has many biochemical applications,^[25]^ including in studies of cell ageing and oxidative stress, in peptide sciences,^[26]^ and in material sciences and organic synthesis.^[27]^ For example, it can increase the permeability of oxygen in water through a polypeptide film, crucial for contact lenses.[^28^, ^29^] Traditionally, the oxidation of sulfides is done with peroxides, but this method often suffers from over‐oxidation to the corresponding sulfones.^[30]^ Using singlet oxygen as a green oxidant solves this selectivity issue, but the reaction conditions are not convenient to scale due to limitations associated with mass transfer and light attenuation.^[31]^ To overcome these challenges, the use of micro‐flow chemistry presents itself as an easy strategy to perform this photocatalytic, singlet oxygen mediated reaction.^[32]^ Methylene Blue (MB) is used as photocatalyst, as it is not only able to produce singlet oxygen under low photon energy excitation, but also matches perfectly with the wavelength emitted by the LR305 luminophore in the LSC‐PM (see Figure 1C).

### Energy supply using photovoltaic cells

**Figure 2:**
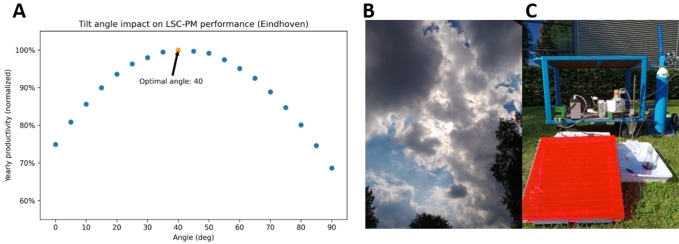
(A) Simulation of the tilt angle impact on yearly productivity of the LSC‐PM mini‐plant. (B) Photographs of the sky conditions at time of the experiment. (C) Experimental setup.

By placing a silicon PV panel at the back side of the LSC‐PM, solar photons with wavelengths that are not absorbed by the LSC‐PM can be collected by the PV and used to power all the peripheral equipment. In this way, maximum use is made of the solar spectrum. For PV arrays with no solar tracking technology, an important parameter to maximize the yearly productivity is the tilt angle, whose dependency with the site latitude is well established.[^33^, ^34^] For LSC‐PM systems, little is known on how the different tilt angles can affect the reactor productivity. The design considerations commonly adopted for PVs cannot be directly translated to the LSC‐PM system as the latter is also capable of harvesting diffuse irradiance.^[35]^ A ray‐tracing Monte‐Carlo algorithm implemented in Python (PvTrace) was used to determine the fate of each photon reaching the LSC.^[36]^ This model was adapted to determine the best fixed tilt angle for the LSC‐PM located in Eindhoven (The Netherlands) (see Figure 2A). A 40° angle for LSC‐PM was determined optimal to maximize the yearly overall number of photons reaching the panel (see Supporting Information).

Regarding the positioning of the PV cell, two options were investigated. Attaching tailor‐made PV cells to the edges of the panel as deployed on standard LSC systems was considered as a first option.^[37]^ This design can be favourable since the LSC‐PM concentrates the photons towards the edges of the lightguide. However, the need for tailored PV cells increases the price for such a device. Furthermore, the small surface area covered by the PV cells, added to the fact that the LSC‐PM reaction channels are meant to gather most of the photons, reduces the attractiveness of this design. The second location investigated places the PV cell directly beneath the reactor. It should be noted that the solar cell and the luminescent dye do not use the same wavelengths and are thus compatible. A PV cell below the reactor could thus process wavelengths between 600–1100 nm, while the more energetic wavelengths are used to catalyse chemical reactions. Moreover, the surface area available beneath the LSC‐PM is more than 10 times greater than the edge surface area (0.221 m^2^ vs. 0.015 m^2^). Hence, for these reasons, the second option was preferred.

We aimed to validate this rationale experimentally by placing a nominal 18 V solar panel below the LSC‐PM and measuring the outdoor energy production levels under a 40° angle approximately. Under sunny conditions, the PV can supply up to 13 W, while, when large clouds pass by, the power production drops to about 4 W.

Next, to estimate if a stand‐alone mini‐plant is possible with a single PV below the LSC‐PM, the energy consumption of the pumping system is measured with a power meter (Brennenstuhl PM231E). Of all the electric components, the HPLC pump is the major power consumer in the mini‐plant and the demand varies with the required flow rate. The power consumption of the system varies between 12–14 W (see Table 1 and Supporting Information for calculations). From these measurements, it is apparent that a single solar panel (0.38×0.51 m^2^) below the reactor is not sufficient to supply a fully autonomous plant of energy. However, positioning the solar panel besides the reactor provides enough power (19 W under sunny conditions and 6 W during cloudy days) and could meet the power demands of the system (Table 1). Yet, by separating the PV panel from the reactor, the entire surface covered would be doubled while producing the same quantity of high‐value chemicals. To cope with this issue, another option would be to increase the surface area of the plant as the peripheral electronic components, including pumps, would remain the same. Consequently, by increasing the size of the reactor and the PV cell underneath, the produced power would surpass the power consumed, as shown in Table 2. In addition, despite the drop in power produced at very cloudy weather, the surplus of energy harvested during sunny moments can be stored in a battery and can, subsequently, act as a buffer to supply this shortfall (sunny conditions are required for approximately 25 % of the time for truly autonomous operation at very cloudy weather).

**Table 1:** Outdoor energy measurements of a solar panel at a 40° angle placed either beneath or next to the LSC‐PM at varying edge emission of the LSC‐PM (EE).

Condition	EE [klux]	*P*_under LSC‐PM_ [W]	*P*_next to LSC‐PM_ [W]	*P*_consumed_ [W]
Sunny	40–44	13	19	14
Very cloudy	16–20	4	6	12

**Table 2:** Extrapolated power consumed and power produced for a 1 m^2^ LSC‐PM system with a 1 m^2^ solar panel mounted directly underneath the reactor.

Condition	EE [klux]	*P*_under LSC‐PM_ [W]	*P*_consumed_ [W]
Sunny	40	63	39
Very cloudy	16	21	29

### Outdoor experiment using the off‐grid solar‐powered autonomous chemical mini‐plant

Next, an outdoor experiment was conducted using the 0.47×0.47 m^2^ LSC‐PM reactor in combination with a 0.38×0.51 m^2^ PV cell behind the reactor under an intermittently cloudy sky (see Figure 2B) in Eindhoven, The Netherlands, from 3 pm to 5 pm on July 15^th^, 2020 (see Supporting Information for a full technical description). Using the Arduino‐based self‐regulating protocol, flow rates of HPLC and MFC were continuously adjusted by the control system to maintain constant conversion. Two additional light sensors were used to record both the direct incident light power and the edge emission of the LSC‐PM at the outdoor location (see Figure 2C). The reaction was conducted for 80 min, with samples taken every 5 minutes and analyzed by HPLC (see experimental section).

**Figure 3:**
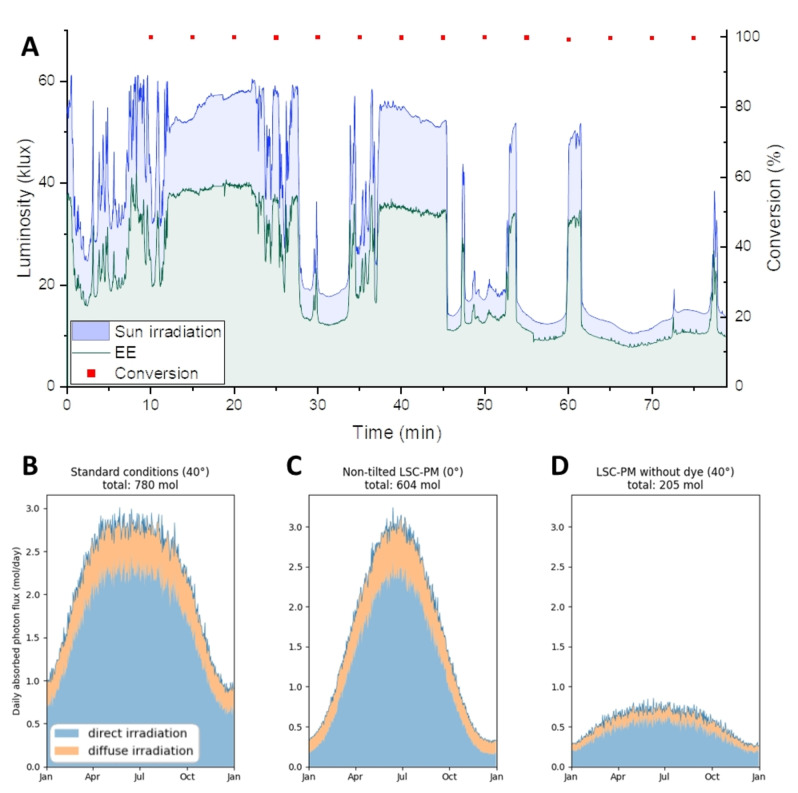
(A) Measurement of direct luminosity (blue area) and edge emission (green area) for 80 min under cloudy conditions 15th of July at 3 pm, and the conversion of L‐methionine to L‐methionine sulfoxide (red dots). Yearly number of photons absorbed by the mini‐plant in Eindhoven in 2020: (B) in normal operating conditions, (C) with no tilt angle and (D) without luminescent dye.

Reduction in luminosity was observed for certain periods during data collection, corresponding to passing clouds (Figure 2B). Despite the fluctuations of irradiation impinging on our reactor, the constant conversion proves the efficiency of the controlling system to autonomously and instantaneously regulate fast changes in light intensity (Figure 3A). The liquid flow rate varied between 2.2 mL min^−1^ and 0.95 mL min^−1^ to ensure that the reaction mixture is receiving sufficient irradiation to reach the targeted conversion. The throughput of the system can experimentally reach up to 17 mmol h^−1^ of *L*‐methionine sulfoxide under strong direct irradiation conditions (i. e., ≈60 klux). Under very low irradiation (i. e., ≈10 klux), the system maintained a throughput of 3 mmol h^−1^. In both scenarios, a high conversion and selectivity for the target compound was ensured. It should be further noted that such high productivities would not be feasible without the light concentrating effect of the LSC‐PM that directs even the diffuse light fraction to the reaction channels. As a consequence, more photons can be harvested compared to transparent reactors, especially at the most challenging reaction conditions, that is, cloudy weather with high fractions of diffuse light (See Simulations and productivity section).

### Simulations and productivity

By simulating the productivity of the reactor in Eindhoven using Monte Carlo ray‐tracing simulations (see Supporting Information), the model can be experimentally validated. For this, the days are assumed to be cloudless throughout the entire year. The total number of photons reaching the reaction mixture during an entire year is depicted in Figure 3B. In comparison to a non‐tilted reactor (Figure 3C), the productivity is more stable throughout the year for the 40° tilted reactor, but with a lower peak productivity in summer. The enhanced productivity due to the addition of the luminescent dye can be seen when comparing the results shown in Figure 3D, where the doped reactor exhibits nearly quadrupled photon absorption as previously reported.^[18]^ With this system, a maximum photon absorption of 2.8 mol day^−1^ can be attained. While our experiment uses photons for chemical conversion, a biphasic mixture is established with oxygen slugs. Since a 1 : 1 volumetric gas‐to‐liquid ratio is required to perform the reaction, the photons reaching the gas will not be absorbed by the photocatalyst to perform the oxidation. Applying the same simulation to the time of the experiment, taking the gaseous volume fraction and the quantum yield of methylene blue^[38]^ (0.39) into account, a maximum productivity of around 55 mmol h^−1^ is predicted. The experiment resulted in a productivity of 17 mmol h^−1^. The difference between the two productivities can be explained by the high conversion target of the reaction. In the experiment, more than 99 % conversion is maintained consistently since conversion was prioritized over productivity. This target was selected as it would be associated with lower and less energy‐demanding downstream purification costs, such as the in‐line catalyst removal by activated charcoal.^[17]^

**Figure 4:**
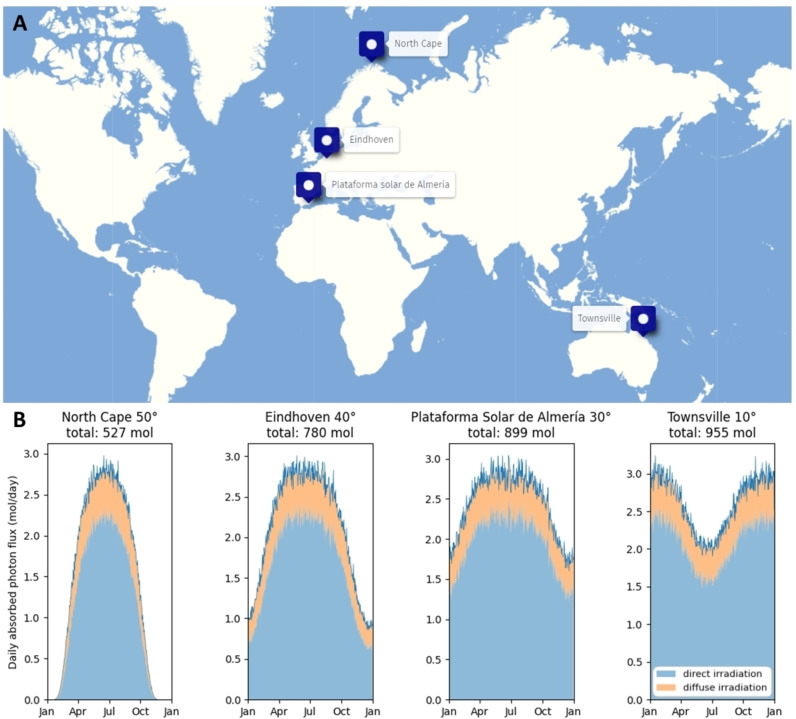
Simulations around the world. (A) Geographical positions of the different locations simulated. Open‐source map from uMap. (B) Simulations of the daily number of photons received in 2020 by the mini‐plant (0.22 m^2^) with optimized tilt angles in North Cape (Norway), Eindhoven (The Netherlands), Almería (Spain), and Townsville (Australia).

Using the same principle, simulations can be used to predict the productivity of the device around the world at different latitudes. Targeting a lower conversion would make the process more photon‐efficient but less relevant to produce clean chemicals (Figure 4A and ESI). Since the final goal of this mini‐plant is to be capable of implementation off‐grid at any location, it is valuable to be able to evaluate the efficiency of a setup anywhere on the globe (or beyond). Based on the spectral and irradiance data of a location (using pvlib^[39]^), we can determine both the optimal tilt angle and give indication of the eventual productivity. An adequate location does not necessarily have to be strongly irradiated by the sun to be suitable, as is normally required for standard silicon PV cells. Thanks to efficient diffuse light collection by the LSC, northern European countries could still use the LSC‐PM device for outdoor chemical production, for example.

Yearly productivity is compared at the optimal angle for the four locations with varying latitudes depicted in Figure 4B; North Cape, Eindhoven, Almeria and Townsville, using a 0.22 m^2^ LSC‐PM reactor. Here, North Cape was chosen because it is the northernmost location in mainland Europe, Eindhoven was chosen to be able to compare our simulations to the outdoor experimental results. In Almería the largest European solar concentrating test and research center, Plataforma Solar de Almería, is located, and Townsville was chosen as a sunny location in the Southern Hemisphere. As expected, higher productivities can be reached in regions closer to the equator, like Townsville (up to 955 mol of absorbed photons per year) or Almería (up to 899 mol of absorbed photons per year). However, the mini‐plant is still capable of reliably producing chemicals from March until November in remote environments like North Cape (up to 527 mol of absorbed photons per year). This demonstrates that the solar‐powered mini‐plant can be deployed and practically used for chemicals production at almost any location where solar energy can be harvested.

Applying this easy‐to‐build system to a known industrial photochemical process, such as Dragoco's rose oxide synthesis,[^40^, ^41^] can justify its use against other options. The key photooxidation can be performed under solar irradiation^[42]^ and would benefit from our LSC‐PM technology. The yearly production of this fragrance molecule is 60–100 tons (390–650 kmol).^[43]^ Thus, by locating the mini‐plant in Townsville, a yearly productivity of 955 mol could be reached. To reach a productivity of 650 kmol year^−1^ of rose oxide, approximately 150 m^2^ of coverage would be needed. Actual industrial solar setups would require around 1900 m^2^ of space deploying parabolic mirrors costing up to 196 € m^−2^;^[43]^ in comparison, the LSC panels required to build the reactor are sold for 99 € m^−2^. Since two LSCs are needed per reactor (see Supporting Information), the price of the light concentrating material reaches 198 € m^−2^. With a similar cost and a smaller surface area required to produce the same quantity of product, the LSC‐PM mini‐plant is actually a promising alternative with more flexible deployment options to industrial photochemical plants. Moreover, as the entire plant is run on solar energy, no energy cost is present in the Operating Expenditures (OPEX), making it a sustainable strategy for future chemical production.

## Conclusion

Herein, we describe the development of an off‐grid, solar‐powered, autonomous chemical mini‐plant for producing fine chemicals under fluctuating solar light irradiation. The reactor consists of a scaled‐up LSC‐PM, which converts direct and diffuse sunlight energy to a wavelength range that matches the absorption spectrum of the photocatalyst and, subsequently, guides this fluorescent light towards embedded reaction channels to drive photochemical transformations. To maintain constant conversion, a calibrated control system assures almost instantaneous adjustments in the chemical feed. This guarantees that the mini‐plant can even be implemented in a rapid weather‐changing environment. To operate the system off‐grid, photovoltaic cells were integrated underneath the reactor to deploy the system in an energy‐neutral manner. Consequently, this mini‐plant can perform photochemical reactions autonomously, even in the absence of a power grid. Furthermore, using our ray‐tracing model, we can predict the optimal array orientation and estimate the productivity of this photochemical mini‐plant based on the local solar spectral data.

Finally, by testing, validating, and extrapolating the behavior of this solar mini‐plant, we demonstrate that it can help with the green production of chemicals, even at remote, off‐grid locations and beyond. We believe this mini‐plant could be especially advantageous for the local production of drugs with a short shelf‐life or to address humanitarian needs, where fast action is often required.

## Conflict of interest

The authors declare no conflict of interest.

## Supporting information

As a service to our authors and readers, this journal provides supporting information supplied by the authors. Such materials are peer reviewed and may be re‐organized for online delivery, but are not copy‐edited or typeset. Technical support issues arising from supporting information (other than missing files) should be addressed to the authors.

Supporting Information
