# Supplementary material for: Development of an Off‐Grid Solar‐Powered Autonomous Chemical Mini‐Plant for Producing Fine Chemicals
Source: ChemSusChem. 2021 Nov 8;14(24):5417–23. doi: 10.1002/cssc.202102011 (PMC9298775; doi:10.1002/cssc.202102011)
Supplement: Supplementary file 1 — Supporting Information [file CSSC-14-5417-s001.pdf]

ChemSusChem

Supporting Information

Development of an Off-Grid Solar-Powered Autonomous Chemical Mini-Plant for Producing Fine Chemicals

Tom M. Masson+, Stefan D. A. Zondag+, Koen P. L. Kuijpers, Dario Cambié, Michael G. Debije, and Timothy Noël*© 2021 The Authors. ChemSusChem published by Wiley-VCH GmbH. This is an open access article under the terms of the Creative Commons Attribution License, which permits use, distribution and reproduction in any medium, provided the original work is properly cited.

Contents

Energy supply 1
Solution and device preparation 1
Control system 2
HPLC analysis 2
Reactor panel 2
Setup calibration and correlation between edge emission and chemical conversion 3
Stability test under LED irradiation 6
Energy consumption of the system 6
Monte Carlo ray-tracing simulations 8
Notes and references 9

Energy supply

The 20 000 mAh battery (Portable Solar Generator Power Station UPS A380, purchased from Banggood) provides a power buffer with fluctuating energy collection and is capable of converting 18 V DC power to 5 V DC, 12 V DC, and to 230 V AC. The last feature is convenient to connect the power supplies of the HPLC pump and the mass flow controller (MFC). The battery is charged via a PV panel (0.38×0.51 m^2) connected in derivation to a voltmeter and an ammeter to measure in real time the power produced.

Solution and device preparation

L-methionine (7.45 g, 50 mmol, Fluorochem) and Methylene Blue (160 mg, 0.5 mmol, Merck) are dissolved in 500 mL of distilled water. The solution and the tubing of the system are covered by aluminum foil, so the irradiation of the reaction mixture occurs only inside of the LSC-PM. The oxygen and the reagent feed are linked by a Y-mixer before entering the LSC-PM. Oxygen is provided via a mass flow controller (Bronkhorst, O_2, max. 25 mL·min^{-1}). The liquid is pumped by an HPLC pump (Knauer Azura P4.1S). The pressure is controlled using a pressure sensor purchased from Huba control. Typically, a bubbly flow is observed at the beginning of the reactor, after which the flow pattern changes to a slug flow. The flow enters the channel closest to the edge emission sensor, then it skips a channel until the other end of the reactor is reached. After this it returns in the same fashion and finally the flow leaves the reactor from the channel second closest to the edge emission sensor. After the flow passed the back pressure regulator (IDEX-HS, P-762, 75 psi), it is either collected in an Erlenmeyer flask or a sample is taken. Analysis is performed using HPLC.

Control system

The HPLC pump and the MFC are controlled by an Arduino Uno programmable board and components. The luminosity emitted by the edge of the panel is measured and sent to the board by a light sensor (Adafruit TSL2591). The volumetric gas-to-liquid ratio is fixed at 1:1 (5:1 at ambient pressure) since a high concentration of oxygen is required due to the highly mass transfer limited character of the reaction.

HPLC analysis

Analysis of the starting material and product, L-methionine and L-methionine oxide, respectively, is performed via high-performance liquid chromatography (HPLC). A Nucleosil® 100-5 C18 column (250 mm length, 4.6 mm internal diameter) obtained from Machery-Nagel is used (part number: 720014.46). Two eluent solutions are prepared: firstly, 0.1 w% trifluoroacetic acid (TFA) in H_2O, and secondly 0.1 w% TFA in acetonitrile. A total flowrate of 1.5 mL·min^{-1} is set with 95% of the water phase and 5% of the acetonitrile phase. A Shimadzu SPD-M20A Diode Array Detector is used at a wavelength of 210 nm to analyse the product (t_r = 2.1 min) and starting material (t_r = 3.8 min).

Reactor panel

In this work, a polymethylmethacrylate (PMMA) lightguide is used. A perfluoroalkoxy alkane (PFA) based capillary was inserted in PMMA sheets, so both chemical and mechanical stabilities were improved. The PMMA material allows scaling-up of the LSC-PMs and, therefore, more productive sunlight driven photocatalytic reactions are possible.

Half a cylinder was drilled out of 4 mm thick PMMA plates doped with 115 ppm of BASF Lumogen F Red 305. Then PFA tubing matching the dimension of the drilled channel (3.2 mm outer diameter, 1.6 mm inner diameter from APT GmbH) was placed inside the groove. A UV curing glue was smeared on the plate and tube, and a second, machined PMMA plate was fit over the tubing. A schematic representation can be seen in Figure 1. This method was used to create a 47×47 cm^2 plate, which included 16 channels with an internal diameter of 1.6 mm (Figure 2).

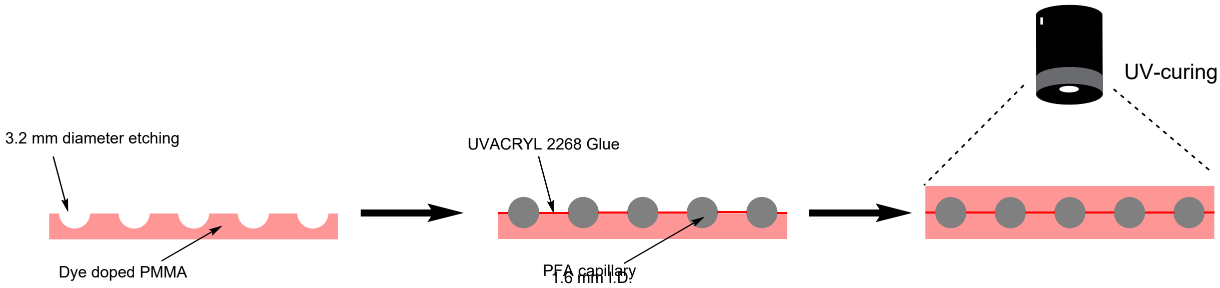

Figure 1 Construction method scaled-up LSC-PM

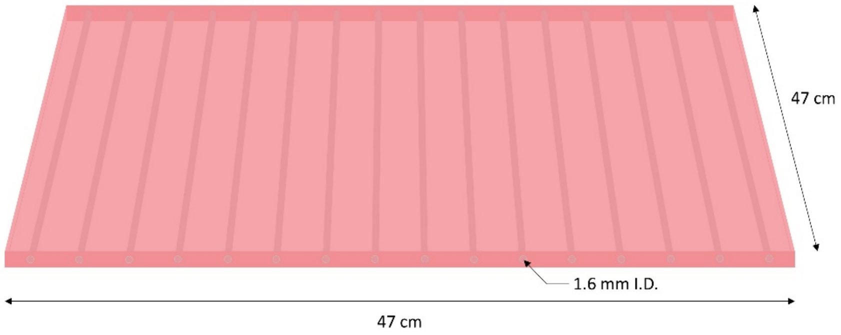

Figure 2 Scaled up LSC-PM

Setup calibration and correlation between edge emission and chemical conversion

To calibrate the system, a homemade light box is used. The light box contains 39 high intensity LED strips (LuxaLight, SMD 5050 RGB, 24 V, 60 LEDs/m, 52 × 52 cm^2) on the ceiling which irradiate the reactor in a homogeneous way. The LED strips are supplied with two power supplies (Velleman LABPS3005DN) that deliver between 16 V and 24 V. Although the intensity and wavelength spectrum from the white LEDs differ from actual solar irradiation, over 90%[1] of the photons that reach the reaction channels are re-emitted by the luminophore and thus have similar wavelength. Therefore, after calibration of a chemical reaction in this Light Box, it is possible to estimate the same reaction conditions under solar irradiation.

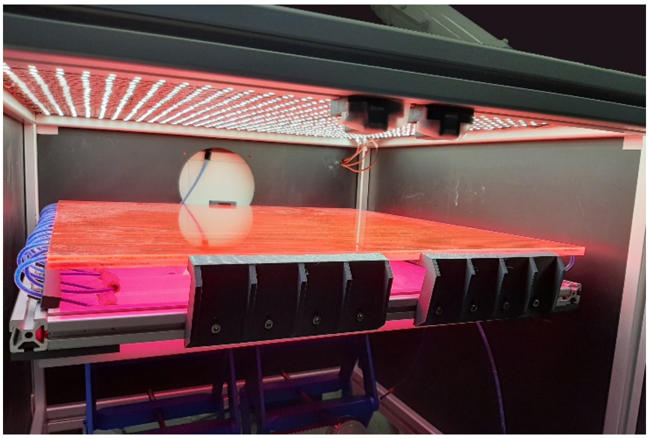

Figure 3 LSC-PM operated with LEDs at 21 V.

In Figure 3 the set-up for the light box measurements is shown. The LED strips can be operated between 16 V and 24 V and deliver a proportional luminosity: note the light sensor at the back of the LSC-PM. The conversion of the reaction is recorded for different flow rates at different LED irradiation intensity using an Adafruit TSL2591 light sensor. In this way a kinetic profile as a function of light intensity may be derived (see Figure 4). The LSC edge emission (EE) is independent of the flow rate used allowing a feedforward system with the edge emission as input parameter (Figure 5). The initial kinetic rates, with up to 90% conversion (obtained from the linear part of the conversion curves), are related to the edge emission. In this way the conversion of L-methionine up to 90% can be predicted. Often a higher purity is desired; for this a linear approximation is made from the 90% conversion point

up to 99.5% or higher. The initial slope of the conversion curve, EE·α, is followed by second slope β. A representation of this method is showed for the 18 V case in Figure 6. The value for β is chosen to be 0.2 mL·min^{-1} for edge emission below 10 klux and 0.5 mL·min^{-1} for edge emission larger or equal to 10 klux. This method resulted in the following equation to calculate the liquid flowrate, Q_L, based on the recorded edge emission:

$$Q_L = \frac{\beta}{X_{target,min} - 0.9 + \frac{0.9 \cdot \beta}{EE \cdot \alpha}} \qquad (1)$$

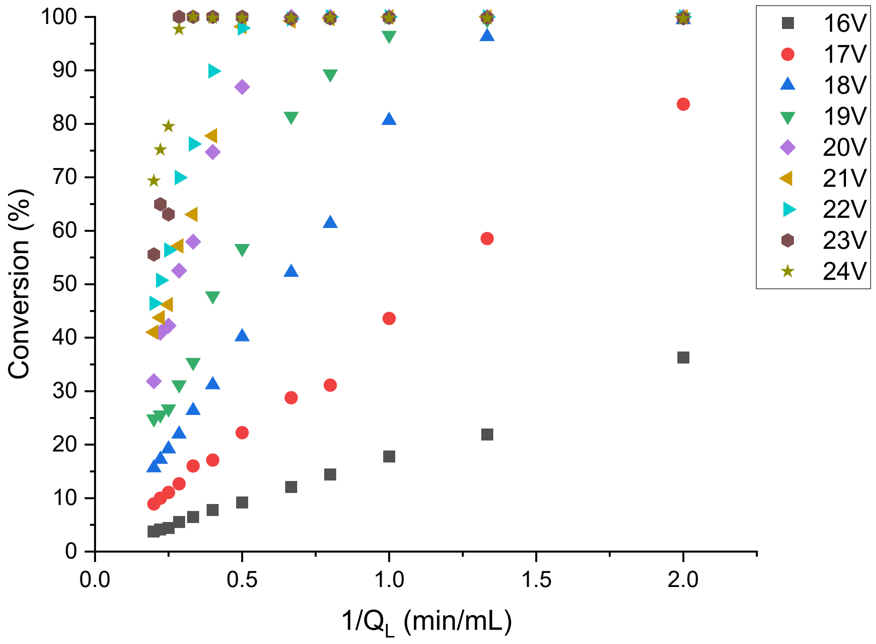

Figure 4 Kinetic profiles for the oxidation of L-methionine at different irradiation intensities, obtained with the Light Box operated at different potentials across the LED array.

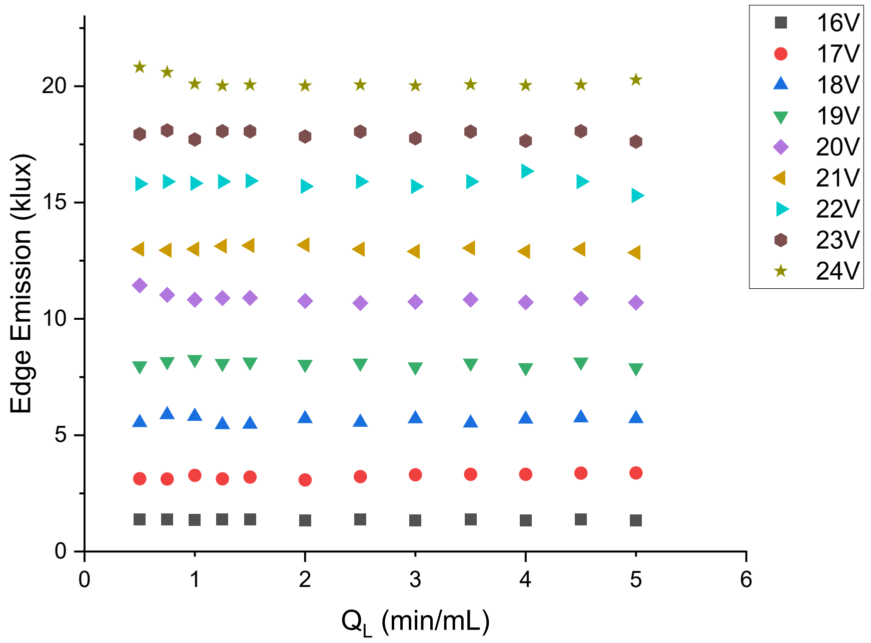

Figure 5 Recorded edge emission (EE) for different set liquid flow rates at different operating voltages of the LED strips.

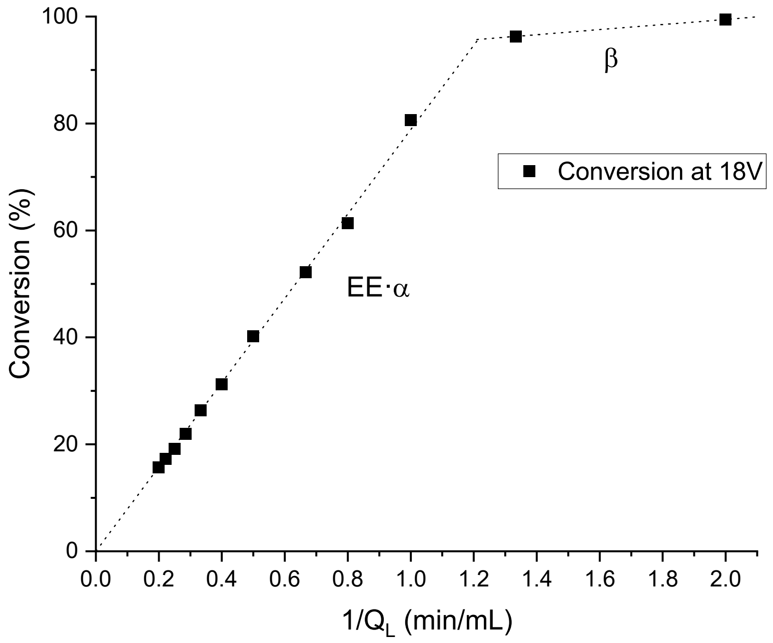

Figure 6 Linearization approach to relate conversion to the desired flowrate.

After the edge emission is recorded, the control box uses equation 1 to calculate which liquid flowrate to set. The gas flowrate is based on the gas-to-liquid 5:1 ratio. The Arduino controller sends signals to the pump and the MFC, which adjust both the gas and liquid flowrates. This feedback process takes slightly more than one millisecond, dictated by the integration time of the light sensor. This fast

response from the control system enables an adjustment of the flow rates almost instantaneous, keeping the conversion constant.

Stability test under LED irradiation

To assess the performance of our feedforward system, the reactor is subjected to fluctuating intensity. The light LED box shown in Figure 3 is used to create a large drop in irradiation. The control system should react to those fluctuations by adapting the flowrates to keep the conversion constant. The conversion measured by HPLC are depicted in Figure 8. For this test, the targeted conversion is set at 95%. With an average conversion of 99.2% and a minimum of 97.6% the control system ensures that the targeted minimal conversion is reached.

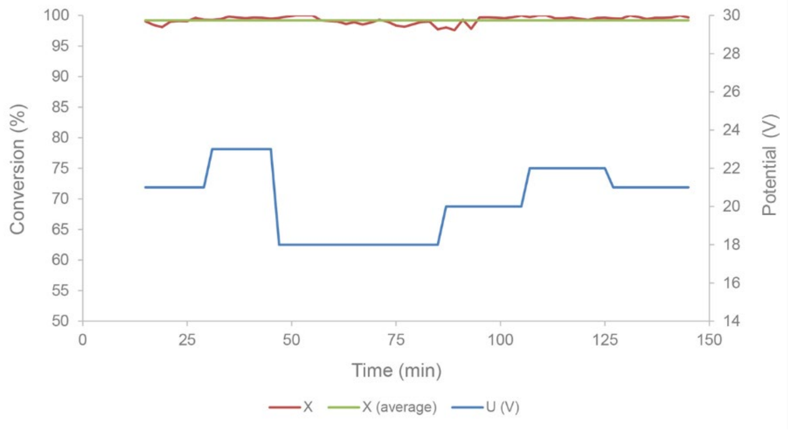

Figure 7 Stability test with feedforward system: conversion of L-methionine (left y axis) at different applied irradiation intensities (right y-axis).

Energy consumption of the system

The power consumed by the MFC and the pump are measured with a power meter (Brennenstuhl PM231E). The control box is estimated to consume 1 W. The actual power consumption is actually significantly lower and it cannot be measured with the power meter used. By using equation 1 to define the flow rate, the power consumed can be linked to the edge emission of the reactor (equations 2 and 3, see Figure 8).

$$P_{consumed} = 3.1657 \cdot Q_L + 6.1143 \quad (2)$$

$$P_{consumed} = 3.1657 \cdot \frac{\beta}{X_{target,min} - 0.9 + \frac{0.9 \cdot \beta}{EE \cdot \alpha}} + 6.1143 \quad (3)$$

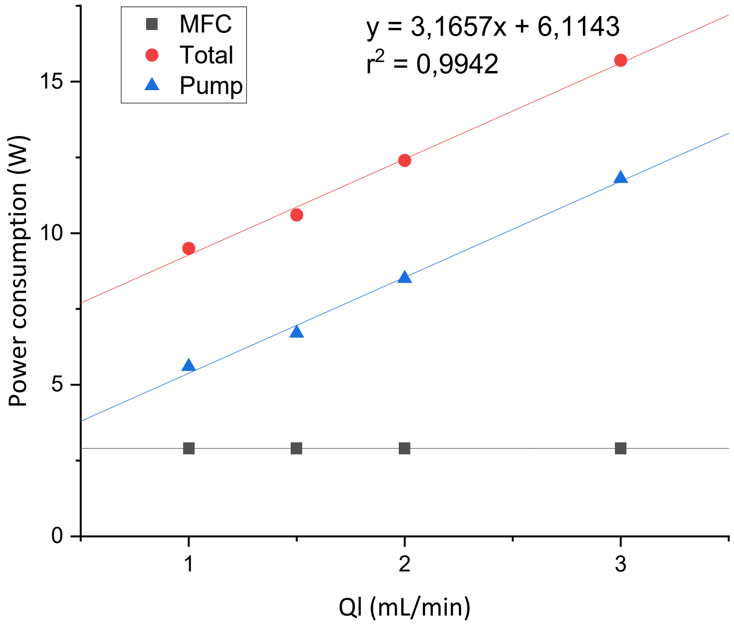

Figure 8 Energy consumption of used equipment: HPLC pump, MFC and the total consumption of HPLC pump + MFC + control box.

To predict the power consumption of the system depending on the solar irradiation we use Equation 3 to link the edge emission of the panel and the power consumption. The targeted conversion is set at 0.99, β is set at 0.3 mL/min and α at $1.486 \cdot 10^{-4}$. Those parameters are set to insure complete conversion at all time. Knowing the edge emission at a certain time, the power required to supply the module can be deduced.

To extrapolate the concept and develop a scaled up version of the module, several adjustments should be made. In order to insure a constant residence time, the flow rates would have to be increased proportionally. Since the power demand of the pump is dependent on the flow rate, the power consumption would rise. It is important to estimate the power required in order to know if a stand alone system is possible.

Since the module is an 0.47 × 0.47 m^2 surface with a 15 mL volume, a 1 × 1 m^2 system would have approximately a volume of 68 mL. To keep the residence time constant inside the channels, the flow rate should be adapted from 2.21 $mL \cdot min^{-1}$ to 10.0 $mL \cdot min^{-1}$ (under high irradiation, at 40 klux). With Equation 2 the power is then estimated at 38.7 W. The power consumption can be evaluated in the same fashion at low irradiation at 16 klux.

To assess if the solar panel under the reactor can supply enough power, the same calculations must be made for the power production. Under sunny conditions (40klux), the solar panel under the LSC-PM produces up to 13.2 W for a surface of 0.38 × 0.51 m^2. Knowing that the electricity production of the panel is correlated with its surface in a linear way, the power produced by a 1 × 1 m^2 panel is estimated at 63 W. Under sunny conditions, the solar panel would be sufficient to power the system. By doing the same calculations under cloudy conditions, it can be noted that the power demand is higher than the power produced. However, the excess of power produced under sunny conditions combined with the battery are more than sufficient to act as a buffer and power the plant when the irradiation is low.

Monte Carlo ray-tracing simulations

An important parameter influencing the LSC-PM reactor performance, especially at higher latitudes, is its tilt angle. Unfortunately, due to the intrinsic differences between LSC-PM and photovoltaic cells working principle, the optimal angle used for the latter cannot be directly applied to the former. In total, four separate locations with different latitudes were investigated: three of them on the northern hemisphere and one on the southern hemisphere (Table 1). To find the optimal tilt angle for these locations, a series of ray-tracing simulation where performed, assuming clear sky conditions, with variable tilt angle (between 0° and 90°) and with reactor azimuth at 180°. The normalized total amount of photons absorbed in the year 2020, simulating 100 photons for every 30 minutes of daylight in the year, yielded the relative productivities are shown in Figure 10.

Table 1 Locations used for simulations with their coordinates and optimal tilt angle.

Location	*Country*	*Latitude*	*Longitude*	*Optimal angle*
North Cape	Norway	70.98	25.98	50°
Eindhoven	The Netherlands	51.44	5.65	40°
Plataforma Solar de Almería	Spain	37.09	-2.36	30°
Townsville	Australia	-19.32	146.76	10°

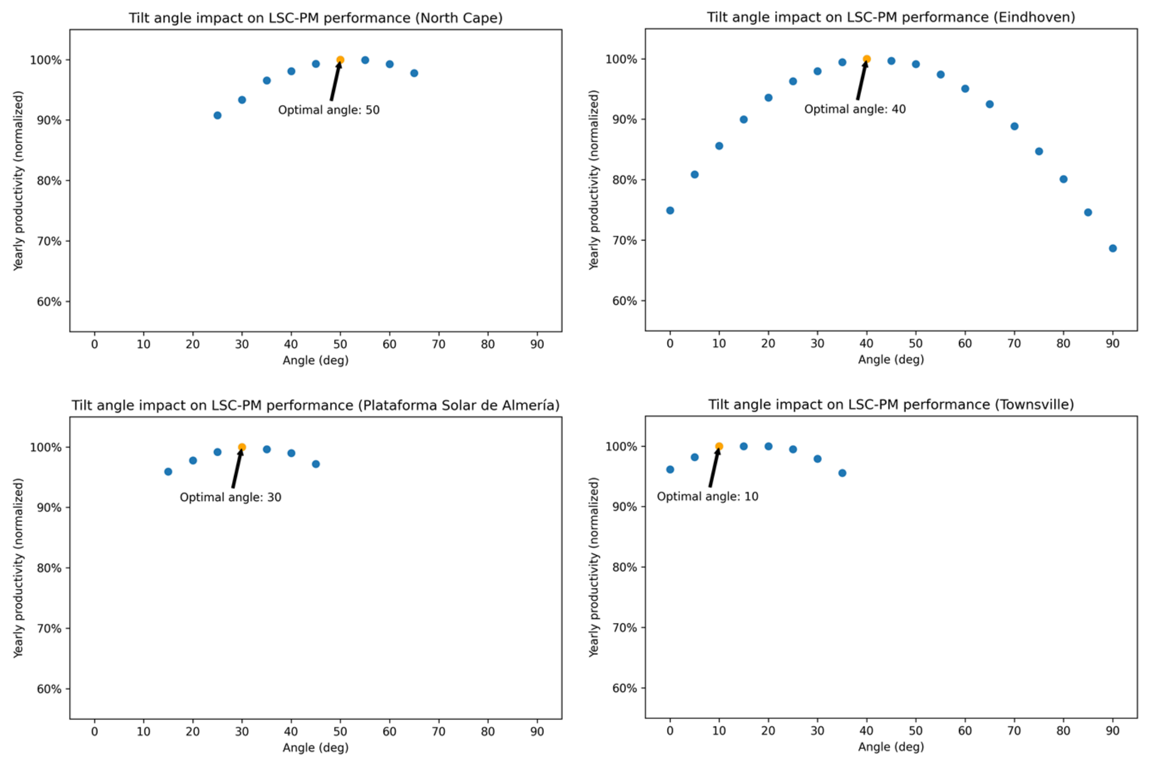

Figure 9 Angle optimization for North Cape, Eindhoven, Plataforma Solar de Almería and Townsville.

To simplify the analysis of the results no weather data was included in the simulations and, as such, the yearly productivity obtained, expressed in mole/reactor-year, represent an ideal "clear sky" scenario. Nevertheless, these results provide a first estimation on order of magnitude of the productivity achievable with an LSC-PM reactor and they allow comparing the reactor performances

in different geographical locations. The yearly productivity simulations included both direct and diffuse irradiance. Direct irradiation originated from the determined location/direction of the sun and the diffuse component was assumed to be isotropic. No ground-reflected irradiance was taken into account. The average internal efficiency of the LSC device, as reported previously,[1] independent of tilt angle, was simulated as 34%.

To validate the simulations with the experimental outdoor results, simulations for the exact day and time in Eindhoven were done (Figure 10). For every half hour, 1000 photons were simulated and the solar spectra at these specific times and location were used to calculate the hourly absorbed photon flux for the mini-plant. This resulted in an average of 282 mmol·h^{-1} for the simulation conditions where the channels were completely filled with reaction mixture. Correction for the 1:1 gas-to-liquid ratio resulted in an absorbed photon flux of 141 mmol·h^{-1}. This is done with the assumption that the linear decrease of reaction mixture causes a linear decrease in absorbed photon flux and neglecting the consumption of the gaseous phase during reaction.

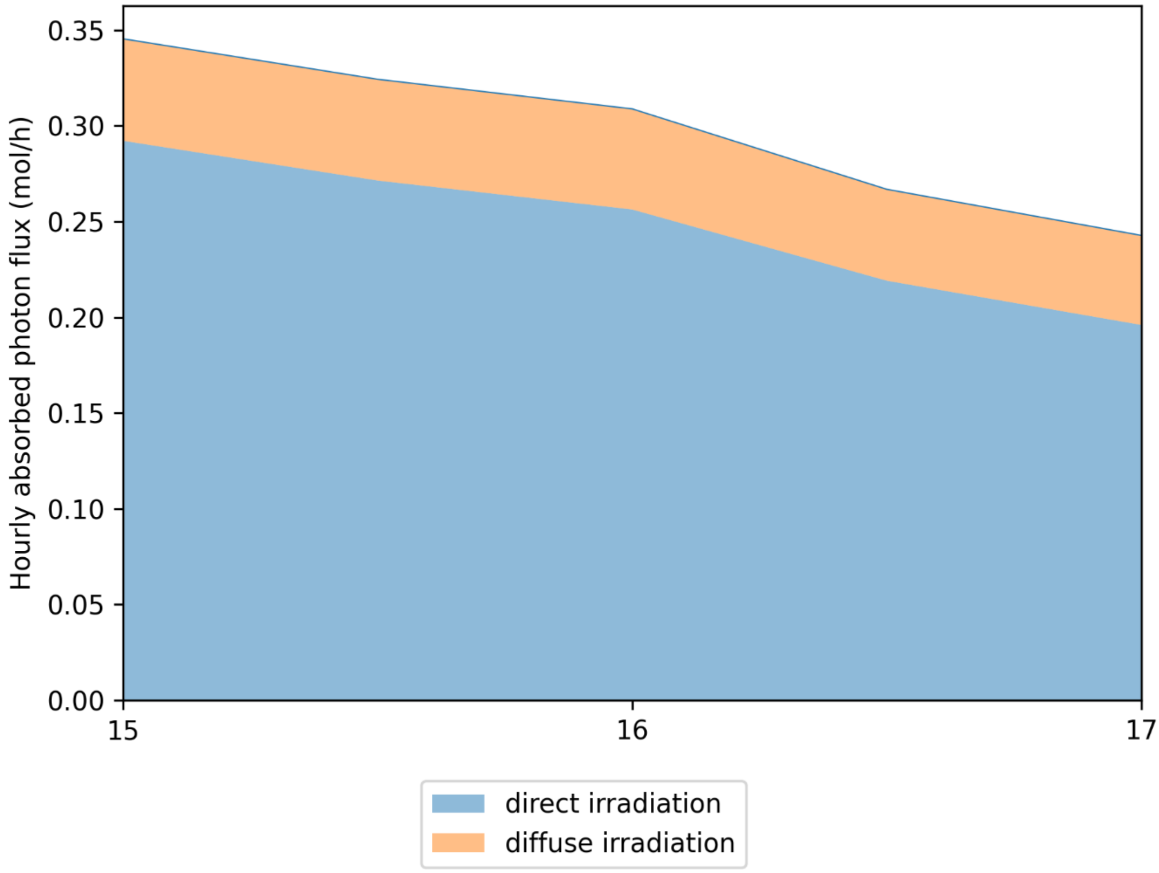

Figure 10 Simulation results of the outdoor experiment the 15th of July 2020, between 3 pm and 5 pm.

Notes and references

1. Cambié, D., Zhao, F., Hessel, V., Debije, M. G. & Noël, T. Every photon counts: Understanding and optimizing photon paths in luminescent solar concentrator-based photomicroreactors (LSC-PMs). *React. Chem. Eng.* **2**, 561–566 (2017).
